# Patient-Representative Cell Line Models in a Heterogeneous Disease: Comparison of Signaling Transduction Pathway Activity Between Ovarian Cancer Cell Lines and Ovarian Cancer

**DOI:** 10.3390/cancers16234041

**Published:** 2024-12-02

**Authors:** Cynthia S. E. Hendrikse, Pauline M. M. Theelen, Wim Verhaegh, Sandrina Lambrechts, Ruud L. M. Bekkers, Anja van de Stolpe, Jurgen M. J. Piek

**Affiliations:** 1 Department of Gynecology and Obstetrics and Catharina Cancer Institute, Catharina Hospital, 5623 EJ Eindhoven, The Netherlands; 2 GROW School for Oncology and Reproduction, Maastricht University, 6229 ER Maastricht, The Netherlands; 3 Philips Research, 5656 AE Eindhoven, The Netherlands; 4 Department of Gynecology and Obstetrics, Maastricht University Hospital, 6229 HX Maastricht, The Netherlands; 5 Department of Gynecology, Radboudumc, 6525 GA Nijmegen, The Netherlands; 6 Drug Companion Diagnostics Company—Therapeutics (DCDC-Tx), 5263 EM Vught, The Netherlands

**Keywords:** ovarian cancer, ovarian carcinoma, cell line, signal transduction pathway, cluster analysis, drug development

## Abstract

**Background/Objectives**: Advances in treatment options have barely improved the prognosis of ovarian carcinoma (OC) in recent decades. The inherent heterogeneity of OC underlies challenges in treatment (development) and patient stratification. One hurdle for effective drug development is the lack of patient-representative disease models available for preclinical drug research. Based on quantitative measurement of signal transduction pathway (STP) activity in cell lines, we aimed to identify cell line models that better mirror the different clinical subtypes of OC. **Methods**: The activity of seven oncogenic STPs (signal transduction pathways) was determined by previously described STP technology using transcriptome data from untreated OC cell lines available in the GEO database. Hierarchal clustering of cell lines was performed based on STP profiles. Associations between cell line histology (original tumor), cluster, and STP profiles were analyzed. Subsequently, STP profiles of clinical OC tissue samples were matched with OC cell lines. **Results**: Cell line search resulted in 80 cell line transcriptome data from 23 GEO datasets, with 51 unique cell lines. These cell lines were derived from eight different histological OC subtypes (as determined for the primary tumor). Clustering revealed seven clusters with unique STP profiles. When borderline tumors (n = 6), high-grade serous (n = 51) and low-grade (n = 31) OC were matched with cell lines, twelve different cell lines were identified as potentially patient-representative OC cell line models. **Conclusions**: Based on STP activity, we identified twelve different cell lines that were the most representative of the common subtypes of OC. These findings are important to improve drug development for OC.

## Simple Summary

Ovarian cancer is difficult to treat because there are several subtypes that differ on the cell level and in their physical characteristics. Cells in the laboratory are used to test the success of a treatment before introducing it in clinical practice. However, these cell line models often fail to accurately represent real-life ovarian cancer tumors. Cells contain a communication network with ‘’chains’’ called signal transduction pathways. These pathways control the cell’s behavior. Aberrant activity in these pathways can cause disbalance in cell’s processes, like cell multiplication and cell death. Ovarian cancer could be represented more accurately by mirroring its behavior through pathway activity. We identified 12 cell lines that had similar pathways compared to an actual patient’s ovarian cancer tissue samples. This could improve the development of promising new treatments that are more effective in patients, making research results more reliable and potentially benefiting future ovarian cancer therapies.

## 1. Introduction

Ovarian carcinoma (OC) has a dismal prognosis, due to the high recurrence rate and limited treatment options. This leads to a 5-year survival of only 23–31% for advanced-stage OC [1,2]. Over the past few years, alternative treatment strategies have been investigated and implemented, such as immune and targeted therapy. However, survival rates have barely improved, showing the difficulty and the complexity of treating OC [2,3].

The development of new treatments is often initiated in pre-clinical studies using cell line models of a representative type of cancer. Unfortunately, preclinical effective treatments often fail to translate to the in vivo situation [4]. One of the key challenges is to replicate in vivo circumstances accurately in the lab [4,5]. OC is a heterogenous disease with multiple subtypes, such as serous, endometrioid (EOC), clear-cell (OCCC) and mucinous OC (MOC), which can further be classified into low- and high-grade OC [3]. Each subtype differs not only in clinical presentation and histologically, but also genomically [6]. One way to phenotypically characterize this heterogeneity is to determine the activity of signal transduction pathways (STPs).

The main functions of STPS are regulation of cell proliferation, differentiation, migration, and cell death [7,8]. Malignant tumors, including OC, arise from dysregulated activity of STPs involved in oncogenesis [8]. Aberrant STP activity may be the direct consequence of genomic alterations, but is also influenced by other factors [8,9]. Previous studies established a clinical benefit for targeted therapy of only 11% and no difference in progression-free survival, when treatment choice was based on genomic alterations [10,11]. The low response rate indicates that the significance of a genomic alteration is overestimated and appears to be an insufficient representation of the actual signaling status in the tumor.

Therefore, it seems pivotal to investigate the effectiveness of drugs on a patient-representative cell line model before introduction into clinical practice. Factors in the tumor micro-environment, such as hormones, immune cells and growth factors, influence STP activity as well [8]. These factors can be easily mimicked in cell culture, on the premise that their effect on the cancer cells can be measured [4]. During the past decade, a robust messenger RNA (mRNA)-based technology to quantitatively measure the activity of the clinically relevant STPs on any cell-containing sample was developed. This technology is based on mathematical Bayesian network STP models that infer STP activity from target gene mRNA measurements [7,12]. This technology is a useful tool in preclinical drug research to improve cell line-based OC models with respect to mimicking clinical OC.

To identify the most patient-representative cell line-based OC models for the different OC subtypes, we compared STP activity between OC cell lines and clinical OC tumor samples.

## 2. Materials and Methods

### 2.1. Datasets

The Gene Expression Omnibus (GEO) was searched via https://www.ncbi.nlm.nih.gov/geo/ (accessed on 5 December 2023) for datasets containing Affymetrix data of ovarian cancer cell lines [13]. We limited our search to datasets containing Affymetrix HG-U133Plus2.0 data [14]. Only untreated cell line samples were analyzed. Cell line samples were excluded in the case they were contaminated with another cell type. In case multiple eligible samples of the same cell line were used in the same dataset, e.g., three replicates, the mean STP activity was calculated.

Baseline characteristics of each individual cell line, such as origin and histopathological subtype, were retrieved from the online database Cellosaurus (RRID:SCR_013869P). Additional information on in vitro culture conditions was retrieved from the publications associated with the respective GEO dataset.

### 2.2. Signal Transduction Pathway (STP) Technology

STP technology has been developed over the past decade at Philips Research. It enables quantitative measurement of activity of the clinically relevant STPs simultaneously on any cell or tissue sample, based on transcriptome (RNA) analysis [14]. This results in a series of STP activity scores, together called the STP profile. The development and validation of mRNA-based assays for measuring activity of ten STPs have been described before [7,12]. In brief, for each STP, activity is measured by a Bayesian modelling-based probabilistic computational model that infers an STP activity score from mRNA levels of a defined set of high evidence direct target genes of the STP-associated transcription factor. Approximately 25 to 35 target genes per STP were selected based on scientific literature to create the Bayesian computational model [7,12]. The specific set of target genes selected for each STP can be found in earlier publications [14,15,16,17,18,19].

Publicly available Affymetrix expression microarray data were downloaded from the GEO database. Data from eligible OC cell lines were analyzed to assess the functional STP activity of the hormonal STPs: Estrogen Receptor (ER) and Androgen Receptor (AR); the growth-factor STPs: Phosphoinositide-3-Kinase (PI3K), Mitogen-Activated Protein Kinase (MAPK) and Janus Kinase- signal transducer and activator of transcription 3 (JAK-STAT3); the immune STPs: Nuclear Factor kappa-light-chain-enhancer of activated B-cells (NF-κB); the developmental STP Hedgehog (HH), Transforming Growth Factor Beta (TGF-β), Notch and Wnt. Since the STP activity of growth-factor driven pathways can be influenced by cell culture conditions (e.g., the amount of fetal calf serum), the MAPK, PI3K, JAK-STAT3 pathways were excluded from analysis [20].

STP activity scores were presented on a normalized (0–100) scale. These scores represent the odds of an STP being active. A score of 0 corresponds to the odds of an STP being inactive and a score of 100 corresponds to the odds of an active STP. Each STP has a specific range of activity (minimum–maximum activity) on this scale. This means that in the interpretation of scores, it is important to consider that STP scores of different pathways cannot be compared directly. The reason for this is that some pathways have a smaller activity range, where small differences are possibly more meaningful compared to pathways with a larger range.

### 2.3. Clustering Analysis

Firstly, hierarchical clustering was performed to analyze the formed clusters of the OC cell lines. The results of hierarchal clustering were presented in a dendrogram for visualization purposes. Secondly, we conducted clustering analysis based on STP activity scores of the eligible cell lines using the least squares method. The STP profile of each cell line was compared to predefined clusters as defined in the clinical study by Tothill et al. [21,22]. These predefined clusters are six molecular subtypes based on gene expression profiling. Methods, results and conclusion from this additional analysis are described in Supplementary File S1.

### 2.4. Matching STP Profiles of Cell Line Models to Patient OC Samples

STP scores from clinical tissue samples of a different OC histology were available from previous studies from our research group [23,24]. Tissue samples included high-grade serous ovarian carcinoma (HGSOC), primary serous borderline ovarian tumors (SBOT) and low-grade ovarian carcinoma (LGOC) tumor tissue and underwent nearest neighbor analysis. The LGOC tissue sample histology included primary or recurrent low-grade serous OC (LGSOC), EOC and MOC. Taking the incidence of the different subtypes into account, this tissue sample group is representative of the main histological subtypes of OC [6]. Data were presented in radar maps for visualization purposes.

### 2.5. Statistical Analysis

Clusters and subgroups were compared using the Kruskal–Wallis test in statistical analysis. A *p*-value of *p* < 0.05 was considered statistically significant. All analyses were performed using R Statistical Software (version 2.1.2; R Foundation for Statistical Computing, Vienna, Austria).

## 3. Results

### 3.1. Datasets and Cell Line Characteristics

The GEO search resulted in 23 eligible datasets (Figure S3) [25,26,27,28,29,30,31,32,33,34,35,36,37,38,39,40,41,42,43,44]. The datasets contained a total of 555 cell line samples. After excluding cell line models that were labeled as contaminated with other cancer type cells, as similar controls, as non-ovarian origin, or as treated samples, data of 80 cell line samples were included in the STP analysis. The 80 cell line samples belonged to 51 unique cell lines. Among the cell line samples, there were eight different subtypes of tumor histology of origin. Baseline characteristics of the included cell lines are described in Table 1.

### 3.2. Hierarchal Clustering of Ovarian Carcinoma Cell Lines

**Figure 1:**
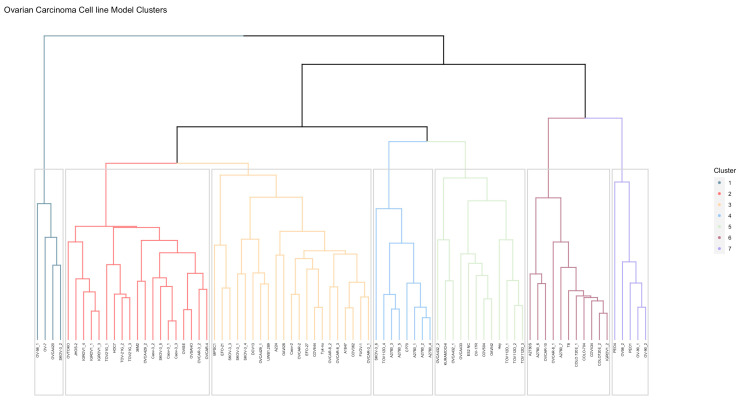
Hierarchal clustering of 80 ovarian carcinoma cell lines based on signal transduction pathway activity, resulting in 7 clusters. Each color represents one cluster. Clusters were characterized with the following STP activity: cluster 1 (n = 4) high AR and high ER and TGF-β STP activity. Cluster 2 (n = 19): relatively low AR and ER STP activity. Cluster 3 (n = 21): relatively high AR and low HH pathway. Cluster 4 (n = 8): high HH STP activity and low NF-κB STP activity. Cluster 5 (n = 12): high Wnt and high HH STP activity. Cluster 6 (n = 11): low TGF-β, Notch and Wnt STP activity. Cluster 7 (n = 5): high AR, ER and low HH STP activity.

Hierarchal clustering of the 80 included samples resulted in seven clusters, as visualized in the dendrogram (Figure 1). Clusters 1, 4, 5, 6 and 7 had distinct pathway characteristics. Cluster 1 (n = 4) is characterized by significantly active hormonal pathways: high AR (*p* < 0.01) and high ER STP activity (*p* < 0.01, compared to cluster 2 to 6), as well as a very high TGF-β pathway (*p* < 0.01) compared to the other clusters. Cluster 2 (n = 19) contained cell lines with relatively low AR and ER STP activity (*p* < 0.05 compared to cluster 1,3 and 7). Cluster 3 (n = 21) distinguished itself by a relatively high AR (*p* < 0.001 compared to cluster 2, 4 and 6) and TGF-β pathway activity compared to other clusters (*p* < 0.01 compared to cluster 2, 4, 6 and 7). Cluster 4 (n = 8) had a significantly high HH STP activity compared to all clusters (*p* < 0.01), but the lowest NF-κB STP activity (*p* < 0.01 compared to cluster 1, 2, 3 and 7). Cluster 5 (n = 12) was defined by a relatively high Wnt (*p* < 0.01 compared to cluster 1, 2, 3, 4 and 6) and high HH STP activity (*p* < 0.05 compared to cluster 1, 2 and 3). Cluster 6 (n = 11) depicted significantly low STP activity among all clusters in the Notch (*p* < 0.001) and Wnt (*p* < 0.05) pathways. This cluster also had a relatively low AR (*p* < 0.001 compared to cluster 1, 3, 5 and 7) and low NF-κB (*p* < 0.05 compared to cluster 1, 2, 3 and 7). Cluster 7 (n = 5) was characterized by a relatively high ER STP activity (*p* < 0.01 compared to cluster 2, 3, 4, 5 and 6) (Figures S4 and S5). There was no association between cluster and cell line histology of origin.

### 3.3. Cell Line Histology of Origin

The included cell lines in this study were derived from a total of eight different subtypes of OC (Table 1). The cell lines were grouped by cell line histology of origin for analysis. Cell lines derived from EOC showed the most distinct STP profile with significantly low AR, NF-κB and a high HH activity. Cell lines derived from HGSOC were distinctively low in AR STP activity. Cell lines derived from OC, with an unknown histological subtype, were high in both the hormonal pathways AR and ER. Ovarian serous adenocarcinoma (OCS), with unspecified histopathological grade, was characterized by high AR STP activity. Possibly because of single samples, there were no significant differences found for LGSOC and ovarian small cell carcinoma, hypercalcemic subtype (OSCC) (Table 2, Figure S6).

### 3.4. Cross-Laboratory Analysis of the Same Cell Lines: Analysis of Variation in STP Profile

**Figure 2:**
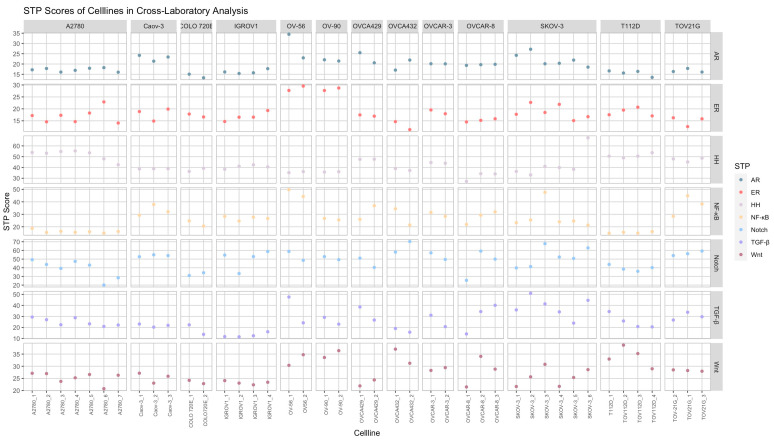
Cell lines included in cross-laboratory analysis, grouped by cell line type. AR = Androgen Receptor Pathway, ER = Estrogen Receptor pathway, HH = Hedgehog pathway, NF-κB = Nuclear factor kappa B pathway; TGF-β = Transforming Growth Factor-Bèta pathway.

Data were available from 13 different cell lines that were produced in different laboratories. Eight of these cell lines fell in two or more of the seven clusters that resulted from hierarchal clustering (Figure 1). Individual STP scores of same cell lines are presented in Figure 2. To explore the factors that cause heterogeneity in STP activity among cell lines, growth protocols and laboratory circumstances were retrieved from the respective papers and GEO database. The majority of the cell lines were cultured in RMPI 1640 with 10% fetal bovine serum. Media were sometimes supplemented with NaHCO_3_, glutamine, non-essential amino acids or PenStrep. Most of the protocols did not describe cell line passage numbers. In this small number of cell lines, no direct relations between STP activity and specific cell culture protocols were found.

### 3.5. Matching OC Cell Lines and OC Tissue Samples

**Figure 3:**
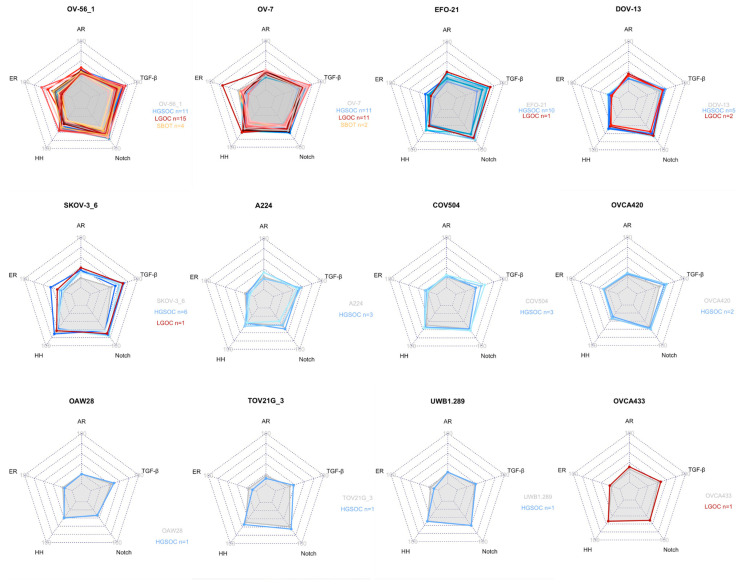
Radar maps of each cell line that were matched to patient tissue samples of serous borderline ovarian tumors (SBOT (yellow)), high-grade serous ovarian carcinoma (HGSOC (blue)) and low-grade ovarian carcinomas (LGOC (red)). In Table 3, the proportions of the tissue histology per cell line are described. Patients’ samples were matched by the AR (=Androgen Receptor), ER (=Estrogen Receptor), HH (=Hedgehog), Notch, and TGF-β (=Transforming Growth Factor Bèta) signal transduction pathways (STPs). The grey area represents the reference STP activity of the respective cell line. The colored lines represent individual patient samples of the tumor tissue.

STP activity scores of 87 tissue samples were available for analysis: 6 SBOT, 50 HGSOC and 30 LGOC tumor tissue samples. LGOC tumor samples included primary or recurrent tumors, including EOC (n = 3), MOC (n = 1) and LGSOC (n = 26). Out of the 80 cell lines, 12 matched to one or more OC subtypes using nearest neighbor analysis. With a total of 26 patient samples matching, cell line OV-56_1 had the highest number of pairings. For the cell lines OVCA433, UWB1.289, OAW28 and TOV21G_3, each matched with one tissue sample (Figure 3).

Next, the cell lines were analyzed by histological subtype of clinical tumor tissue. Cell lines were grouped by matched SBOT, HGSOC and LGOC tissue to explore the representability of the cell lines for different clinical OC subtypes. HGSOC samples were matched to a total of 11 different cell lines. In total, 22% of HGSOC samples were matched with cell line OV-7, followed by EFO-21 (20%) and OV-56_1 (14%). Cell lines OVCA420, UWB1.289, OAW28, COV504, A224 and TOV21G_3 only matched with HGSOC samples. The LGOC samples were matched to six different cell lines: DOV-13, SKOV-3_6, EFO-21, OV-56_1, OV433 and OV-7. Half of the LGOC samples were matched to OV_56_1, followed by 46% matched to OV-7. OV433 was a LGOC unique cell line. SBOT was matched to OV_56_1 and OV-7 (Table 3).

Based on the proportions of the matched tumor tissue histology, EFO-21 and SKOV-3 are the cell lines most representative for HGSOC. Cell lines OV-56 and OV-7 are the most representative for LGOC based on STP activity. Table 3 further describes a recommendation for representative cell lines for both LGOC and HGSOC from most to least recommended.

Lastly, we explored if there was an association between the above identified cell lines that were the most representative for clinical OC, and the clusters that resulted from cell line hierarchal clustering. The 12 representative cell lines were included in 5 out of 7 clusters (Figure 1). Five of these cell lines were found in cluster 3. Three cell lines were included in cluster 1, characterized by high AR, ER and TGF-β STP activity. Furthermore, there were two matched cell lines in cluster 5, the HH and Wnt active cluster. Both cluster 2 and 4 contained one matched cell line. The majority of tumor samples matched to the respective cell lines were included in cluster 1 (n = 52) and cluster 3 (n = 23). In cluster 1, the cell lines are included that matched with all SBOT tissue samples and the majority of the LGOC tissue samples, indicating that this cluster of the cell lines is potentially the most representative for LGOC and tumors with low malignant potential. Cluster 3 contains cell lines with a preference for HGSOC. This indicates that the cell lines included in these clusters are potentially the most representative for the respective subtypes of OC.

## 4. Discussion

In our study, 12 cell line models are identified based on STP profiling as representatives for the majority of OC subtypes. Furthermore, we detected that OC cell lines can be clustered in seven different clusters based on STP activity. These clusters—containing cell lines with matched OC tissue samples—are possibly also of use as representable OC cell lines in pre-clinical research.

A recent study of Mccabe et al. produced a cell line panel including recommendations for representable cell lines for four major histological subtypes of OC based on their gene expression profiles [45]. Out of our 12 cell lines based on STP activity in our study, 9 were also investigated by Mccabe et al. Our findings partially align with the recommended two cell lines for HGSOC and one cell line for LGOC. However, the MOC cell line, which was not recommended for use, was found to be a patient-representative HGSOC cell line model in our study. Interestingly, four cell lines identified as OCCC by Mccabe et al. matched with most HGSOC and LGOC samples based on STP activity in our study. Although there were no OCCC tissue samples available to match, the histology of the matched clinical OC samples is surprising, considering only one of these cell lines was originally derived from OCCC. The differences in results can possibly be explained by not taking other important STP components into account. From these findings, it can be concluded that cancer cells undergo substantial genomic alterations during cell culture, resulting in a loss of the phenotypical characteristics of the original histology.

The loss of phenotypical characteristics was confirmed in the observed cross-laboratory STP heterogeneity between the same cell lines, suggesting that different (extrinsic) factors influence the STP activity. Ben-David et al. established that a possible underlying factor for genomic variety is subclonality [46]. Additionally, they found genetic variety to be associated with passage number and culture conditions. Finally, the authors hypothesized de novo variations in gene expression. The cross-laboratory STP heterogeneity adds to these findings and probably mirrors clonal evolution. To exactly mirror individual clonal evolution is infeasible because cancer cell cultures have substantially fewer factors influencing the tumor cells compared to cancer in vivo. With abundantly large number of (unique) interactions on a molecular level and additional clinical differences between patients, it is expected that the clinical OC heterogeneity is even larger than the (cross-)laboratory heterogeneity. Therefore, the use of representable cell lines in preclinical research or even a personalized approach is more warranted.

Our findings may be applied in pre-clinical research, including drug development. Utilizing representative cell line models should make the translation from in vitro to in vivo easier. However, it is unknown if a matched tumor profile based on the phenotype of the cell is sufficient to represent a tumor accurately. Manipulating the STP activity to the desired tumor profile, such as adding TGF-β to the cell medium, and the effects on drug sensitivity should therefore be an area of research. Furthermore, one major hurdle to overcome in drug testing is cellular crosstalk. Taking the PI3K pathway as an example, an extracellular activated PI3K pathway through growth factors in cell culture possibly expresses the same STP activity as a patient’s tumor cross-activated through the MAPK pathway [47]. In theory, a receptor tyrosine kinase antagonist would lower the activity of the PI3K pathway in the first example, but in the second case, the PI3K activity would not change because a downstream inhibitor, such as an AKT or mTOR inhibitor, is necessary to establish the same effect. These different mechanisms should be noted when testing targeted therapy based on STP activity, since possible upstream and downstream targets can show different effects.

To the best of our knowledge, this is the first study to investigate functional STP activity in OC cell lines. Other strengths include the use of the publicly available GEO database and the use of our previous study results to translate our current results for application in clinical practice [23,24]. Limitations of the cell line matching performed in this study include the exclusion of the STPs that are influenced by laboratory conditions (culture medium, percentage of fetal bovine serum etc.), although they might play a major role in the pathophysiology of OC. So far, approximately ten STPs are known to be oncogenic [14]. High MAPK and PI3K STP activity are frequently present in LGSOC and HGSOC, respectively [48,49]. Due to the exclusion of these important oncogenic pathways, accurate matching to the patient’s tumor is suboptimal. Therapies specifically targeting these pathways could be tested in carefully planned studies taking the laboratory circumstances and its possible effect on these STPs into account. Secondly, a significant portion of the cell lines had an unspecified histological subtype (OC, OCS). This could be the reason that no distinct pathways profiles were identified and there was a wide range of activity for some pathways compared to other subtypes (Figure S6). Lastly, it is possible that other representative cell lines were not identified due to the matching method used. The least squares method matched each clinical OC tissue sample to the most matched cell line; however, there are possibly more cell lines that are sufficiently representative, for example the remaining cell lines in the same cluster that did not match with an OC tissue sample.

The effects of treatment on functional STP activity should be further investigated. Most patients who are considered for targeted therapy have recurrent disease. For more accurate cell line representation, it may be beneficial to take the treatment history into account so drug resistance mechanisms can be mimicked. This can be achieved by using platinum-resistant OC cell lines with a similar STP profile. Knowledge on heterogeneity of OC is crucial for these experiments in drug development. A study using single-cell RNA sequencing could give insights into the differences between patients. Additionally, identification of patient-representative OC cell lines for primary and recurrent OC may also be helpful since recurrent OC has more genomic alterations and has a different STP profile [24,50]. The remaining cell lines in the identified clusters may be further investigated in order to find more patient-representative cell line models.

## 5. Conclusions

In conclusion, we identified a selection of cell line models that are most representative for the majority of the high- and low-grade OC subtypes based on functional STP activity. This selection of cell line models might better reflect in vivo tumors and therefore, it is recommended to use specific lines for drug development for specific OC subtypes.

## Figures and Tables

**Table 1:** Baseline characteristics of cell lines included in the analysis. The table contains the GSE number from the Gene Expression Omnibus (GEO). Histology of origin was retrieved from the information provided on the GEO or from the Cellosaurus (knowledge resource on cell lines).

Cell Line.	GSE Dataset	Histology of Origin
A1847	GSE28724	Ovarian carcinoma
A224	GSE120245	Ovarian serous adenocarcinoma
A2780	GSE28724 GSE50831	Endometrioid ovarian carcinoma
A2780CP	GSE23553	Endometrioid ovarian carcinoma
A2780S	GSE23553	Endometrioid ovarian carcinoma
Caov-2	GSE28724	Ovarian carcinoma
Caov-3	GSE28724 GSE50831	High-grade serous ovarian carcinoma
COLO 720E	GSE50831	High-grade serous ovarian carcinoma
COLO 704	GSE50831	High-grade serous ovarian carcinoma
COV362	GSE50831	High-grade serous ovarian carcinoma
COV434	GSE50831	Ovarian small cell carcinoma, hypercalcemic type
COV504	GSE50831	Ovarian carcinoma
COV644	GSE50831	Mucinous ovarian carcinoma
DOV13	GSE28724	Ovarian carcinoma
EFO-21	GSE28724	Ovarian serous cystadenocarcinoma
EFO-27	GSE50831	Mucinous ovarian carcinoma
ES2	GSE106549	Ovarian serous adenocarcinoma
FUOV-1	GSE28724	High grade serous ovarian carcinoma
HOC7	GSE148648	Ovarian serous adenocarcinoma
IGROV1	GSE23553 GSE28724 GSE18680	Endometrioid ovarian carcinoma
JHOS-2	GSE28724	High-grade serous ovarian carcinoma
KURAMOCHI	GSE50831	High-grade serous ovarian carcinoma
MPSC1	GSE148648	Low-grade serous ovarian carcinoma
OAW28	GSE28724	High-grade serous ovarian carcinoma
OAW42	GSE28724	Ovarian serous cystadenocarcinoma
OV-17R	GSE28724	Ovarian carcinoma
OV-56	GSE28724 GSE50831	Ovarian serous adenocarcinoma
OV-7	GSE28724	Ovarian carcinoma
OV-90	GSE28724 GSE50831	Ovarian carcinoma
OVCA420	GSE28724	Ovarian serous adenocarcinoma
OVCA429	GSE28724	Ovarian cystadenocarcinoma
OVCA432	GSE28724	Ovarian serous adenocarcinoma
OVCA433	GSE28724	Ovarian serous adenocarcinoma
OVCAR-10	GSE28724	Ovarian carcinoma
OVCAR-2	GSE28724	Ovarian carcinoma
OVCAR-3	GSE28724 GSE50831	High-grade serous ovarian carcinoma
OVCAR-4	GSE50831	High-grade serous ovarian carcinoma
OVCAR-8	GSE28724 GSE106549	High-grade serous ovarian carcinoma
OVISE	GSE50831	Clear cell ovarian carcinoma
OVSAHO	GSE50831	High-grade serous ovarian carcinoma
OVTOKO	GSE50831	Clear cell ovarian carcinoma
PEO1	GSE28724	Ovarian cystadenocarcinoma
PEO4	GSE22600	Ovarian cystadenocarcinoma
SKOV-3	GSE28724 GSE50831 GSE18680	Ovarian serous cystadenocarcinoma
TOV112D	GSE18680 GSE28724 GSE50831	Endometrioid ovarian carcinoma
T8	GSE23553	Endometrioid ovarian carcinoma
TOV-21G	GSE50831 GSE18680	Clear cell ovarian carcinoma
Tyk-nu	GSE28724	High-grade serous ovarian carcinoma
UWB1.289	GSE28724	Ovarian carcinoma

**Table 2:** Significantly high/low signal transduction pathway activity compared to other histological subtypes. Pathways are characterized as high or low when the pathway activity is significantly different compared to three or more histological subtypes.

	AR	ER	HH	NF-κB	Notch	TGF-β	Wnt
**EOC**	**Low**	**CNBD**	**High**	**Low**	**CNBD**	**CNBD**	**CNBD**
	HGSOC **	OC*	HGSOC ****	HGSOC ****	OCCC *	OC ***	OC ***
	MOC *	-	MOC *	OC ***	OCS **	OCS ***	OCS ***
	OC ****	-	OC *	OCCC ****	-	-	-
	OCS ****	-	OCS ***	OCS ****	-	-	-
**HGSOC**	**Low**	**CNBD**	**CNBD**	**CNBD**	**CNBD**	**CNBD**	**CNBD**
	EOC **	OC*	EOC ****	EOC ****	-	OC ***	OC ***
	MOC *	-	-	OCCC *	-	OCS **	OCS **
	OC *	-	-	-	-	-	-
	OCS **	-	-	-	-	-	-
**LGSOC**	-	-	-	-	-	-	-
**MOC**	**CNBD**	**-**	**CNBD**	-	-	-	-
	EOC *	-	MOC *	-	-	-	-
	HGSOC *	-	-	-	-	-	-
**OC**	**High**	**High**	**CNBD**	**CNBD**	**CNBD**	**CNBD**	**CNBD**
	EOC ****	EOC *	EOC *	EOC ***	-	EOC ***	EOC ***
	HGSOC *	HGSOC *	-	-	-	HGSOC ***	HGSOC ***
	OCCC ***	OCCC *	-	-	-	-	-
**OCCC**	**CNBD**	**CNBD**	**CNBD**	**CNBD**	**CNBD**	**CNBD**	**CNBD**
	OC ***	OC ***	-	EOC ****	EOC *	-	-
	OCS ***	OCS ***	-	-	-	-	-
**OCS**	**High**	**CNBD**	**CNBD**	**CNBD**	**CNBD**	**CNBD**	**CNBD**
	EOC ****	OCCC *	EOC ***	EOC ****	EOC **	EOC ***	EOC ***
	HGSOC **	-	-	-	-	HGSOC **	HGSOC **
	OCCC ***	-	-	-	-	-	-
**OSCC**	-	-	-	-	-	-	-

CNBD = cannot be determined. AR = Androgen Receptor, ER = Estrogen Receptor, HH = Hedgehog, NF-κB = Nuclear factor kappa-light-chain-enhancer of activated B cells, TGF-β = Transforming Growth Factor-Bèta. EOC = Endometroid ovarian carcinoma HGSOC = high-grade serous ovarian carcinoma, LGSOC = low-grade serous ovarian carcinoma, MOC = mucinous ovarian carcinoma, OC = ovarian carcinoma, OCCC = ovarian clear cell carcinoma, OCS = ovarian serous adenocarcinoma; OSCC = ovarian small cell carcinoma, hypercalcemic type. The red indicates significantly high pathway activity, while the blue represents low pathway activity compared to the respective histological subtype. * *p* < 0.05; ** *p* < 0.01; *** *p* < 0.001; **** *p* < 0.0001.

**Table 3:** Cell lines with their corresponding histological subtype of origin, matched to serous borderline ovarian tumor (SBOT), high-grade serous ovarian carcinoma (HGSOC) tumor samples (n = 50), and low-grade ovarian carcinoma (LGOC) tumor samples (n = 30), along with their respective clusters. The table includes a column with a histological subtype each cell line is the most representative for, ranked from first to last choice, with numbers in parentheses denoting the rank.

Cell Line	Celline Histology of Origin	Amount of Matched Tissue Samples	Tumor Tissue Histology (%)	Hierarchal Cluster	Representative For:
DOV-13	Ovarian carcinoma	7	HGSOC (n = 5) 71% LGOC (n = 2) 29%	3	HGSOC (3)
SKOV-3_6	Ovarian serous cystadenocarcinoma	7	HGSOC (n = 6) 86% LGOC (n = 1) 14%	4	HGSOC (2)
OVCA433	Ovarian serous adenocarcinoma	1	LGOC (n = 1) 100%	5	LGOC (3)
EFO-21	Ovarian serous cystadenocarcinoma	11	HGSOC (n = 10) 91% LGOC (n = 1) 9%	3	HGSOC (1)
OVCA420	Ovarian serous adenocarcinoma	2	HGSOC (n = 2) 100%	1	HGSOC (6)
OV-56_1	Ovarian serous adenocarcinoma	26	SBOT (n = 4) 15% HGSOC (n = 7) 27% LGOC (n = 15) 58%	1	LGOC (1)
UWB1.289	Ovarian carcinoma	1	HGSOC (n = 1) 100%	3	HGSOC (7)
OAW28	High-grade serous ovarian carcinoma	1	HGSOC (n = 1) 100%	3	HGSOC (7)
COV504	Ovarian carcinoma	3	HGSOC (n = 3) 100%	5	HGSOC (5)
A224	Ovarian serous carcinoma	3	HGSOC (n = 3) 100%	3	HGSOC (5)
OV-7	Ovarian carcinoma	24	SBOT (n= 2) 8% HGSOC (n = 11) 46% LGOC (n = 11) 46%	1	HGSOC (4)/LGOC (2)
TOV21G_3	Clear cell ovarian carcinoma	1	HGSOC (n = 1) 100%	2	HGSOC (7)

## Data Availability

The raw data used for analysis in this study are openly available in the Gene Expression Omnibus (GEO database). The datasets generated during analysis in the current study will be available upon request.

## Supplementary Materials

The following supporting information can be downloaded at https://www.mdpi.com/article/10.3390/cancers16234041/s1: Supplementary File S1: Analysis by clusters of Tothill et al.; Figure S3: Flowchart of GEO search and included cell lines in analysis.; Figure S4: Clusters of cell lines resulting from hierarchal clustering by signal transduction pathway activity; Figure S5: Signal transduction pathway scores of cell lines grouped by cluster; Figure S6: Comparison of signal transduction pathway activity of cell lines grouped by histology of origin.

## References

[1] Siegel R.L., Miller K.D., Wagle N.S., Jemal A. (2023). Cancer statistics, 2023. CA Cancer J. Clin..

[2] Chandra A., Pius C., Nabeel M., Nair M., Vishwanatha J.K., Ahmad S., Basha R. (2019). Ovarian cancer: Current status and strategies for improving therapeutic outcomes. Cancer Med..

[3] Kossai M., Leary A., Scoazec J.Y., Genestie C. (2018). Ovarian Cancer: A Heterogeneous Disease. Pathobiology.

[4] Mirabelli P., Coppola L., Salvatore M. (2019). Cancer Cell Lines Are Useful Model Systems for Medical Research. Cancers.

[5] Sajjad H., Imtiaz S., Noor T., Siddiqui Y.H., Sajjad A., Zia M. (2021). Cancer models in preclinical research: A chronicle review of advancement in effective cancer research. Anim. Model. Exp. Med..

[6] Coburn S.B., Bray F., Sherman M.E., Trabert B. (2017). International patterns and trends in ovarian cancer incidence, overall and by histologic subtype. Int. J. Cancer.

[7] van de Stolpe A., Verhaegh W., Blay J.Y., Ma C.X., Pauwels P., Pegram M., Prenen H., De Ruysscher D., Saba N.F., Slovin S.F. (2020). RNA Based Approaches to Profile Oncogenic Pathways From Low Quantity Samples to Drive Precision Oncology Strategies. Front. Genet..

[8] Sever R., Brugge J.S. (2015). Signal transduction in cancer. Cold Spring Harb. Perspect. Med..

[9] Nair A., Chauhan P., Saha B., Kubatzky K.F. (2019). Conceptual Evolution of Cell Signaling. Int. J. Mol. Sci..

[10] Le Tourneau C., Delord J.P., Goncalves A., Gavoille C., Dubot C., Isambert N., Campone M., Tredan O., Massiani M.A., Mauborgne C. (2015). Molecularly targeted therapy based on tumour molecular profiling versus conventional therapy for advanced cancer (SHIVA): A multicentre, open-label, proof-of-concept, randomised, controlled phase 2 trial. Lancet Oncol..

[11] Massard C., Michiels S., Ferte C., Le Deley M.C., Lacroix L., Hollebecque A., Verlingue L., Ileana E., Rosellini S., Ammari S. (2017). High-Throughput Genomics and Clinical Outcome in Hard-to-Treat Advanced Cancers: Results of the MOSCATO 01 Trial. Cancer Discov..

[12] Verhaegh W., Van de Stolpe A. (2014). Knowledge-based computational models. Oncotarget.

[13] Gil-Martin M., Pardo B., Barretina-Ginesta M.-P. (2020). Rare ovarian tumours. Other treatments for ovarian cancer. Eur. J. Cancer Suppl..

[14] Verhaegh W., van Ooijen H., Inda M.A., Hatzis P., Versteeg R., Smid M., Martens J., Foekens J., van de Wiel P., Clevers H. (2014). Selection of personalized patient therapy through the use of knowledge-based computational models that identify tumor-driving signal transduction pathways. Cancer Res..

[15] van Ooijen H., Hornsveld M., Dam-de Veen C., Velter R., Dou M., Verhaegh W., Burgering B., van de Stolpe A. (2018). Assessment of Functional Phosphatidylinositol 3-Kinase Pathway Activity in Cancer Tissue Using Forkhead Box-O Target Gene Expression in a Knowledge-Based Computational Model. Am. J. Pathol..

[16] van de Stolpe A., Holtzer L., van Ooijen H., Inda M.A., Verhaegh W. (2019). Enabling precision medicine by unravelling disease pathophysiology: Quantifying signal transduction pathway activity across cell and tissue types. Sci. Rep..

[17] Cante-Barrett K., Holtzer L., van Ooijen H., Hagelaar R., Cordo V., Verhaegh W., van de Stolpe A., Meijerink J.P.P. (2020). A Molecular Test for Quantifying Functional Notch Signaling Pathway Activity in Human Cancer. Cancers.

[18] Holtzer L., Wesseling-Rozendaal Y., Verhaegh W., van de Stolpe A. (2022). Measurement of activity of developmental signal transduction pathways to quantify stem cell pluripotency and phenotypically characterize differentiated cells. Stem Cell Res..

[19] Bouwman W., Verhaegh W., Holtzer L., van de Stolpe A. (2020). Measurement of Cellular Immune Response to Viral Infection and Vaccination. Front. Immunol..

[20] You M., Xie Z., Zhang N., Zhang Y., Xiao D., Liu S., Zhuang W., Li L., Tao Y. (2023). Signaling pathways in cancer metabolism: Mechanisms and therapeutic targets. Signal Transduct. Target. Ther..

[21] Tothill R.W., Tinker A.V., George J., Brown R., Fox S.B., Lade S., Johnson D.S., Trivett M.K., Etemadmoghadam D., Locandro B. (2008). Novel molecular subtypes of serous and endometrioid ovarian cancer linked to clinical outcome. Clin. Cancer Res..

[22] van Lieshout L., van de Stolpe A., van der Ploeg P., Bowtell D., de Hullu J., Piek J. (2020). Signal Transduction Pathway Activity in High-Grade, Serous Ovarian Carcinoma Reveals a More Favorable Prognosis in Tumors with Low PI3K and High NF-kappaB Pathway Activity: A Novel Approach to a Long-Standing Enigma. Cancers.

[23] van Lieshout L., van der Ploeg P., Wesseling-Rozendaal Y., van de Stolpe A., Bosch S., Lentjes-Beer M., Ottenheijm M., Meriaan A., Vos C., de Hullu J. (2021). Survival Is Related to Estrogen Signal Transduction Pathway Activity in Postmenopausal Women Diagnosed with High-Grade Serous Ovarian Carcinoma. Cancers.

[24] Hendrikse C.S.E., van der Ploeg P., van de Kruis N.M.A., Wilting J.H.C., Oosterkamp F., Theelen P.M.M., Lok C.A.R., de Hullu J.A., Smedts H.P.M., Vos M.C. (2023). Functional estrogen receptor signal transduction pathway activity and antihormonal therapy response in low-grade ovarian carcinoma. Cancer.

[25] Tan T.Z., Miow Q.H., Huang R.Y., Wong M.K., Ye J., Lau J.A., Wu M.C., Bin Abdul Hadi L.H., Soong R., Choolani M. (2013). Functional genomics identifies five distinct molecular subtypes with clinical relevance and pathways for growth control in epithelial ovarian cancer. EMBO Mol. Med..

[26] Au-Yeung C.L., Yeung T.L., Achreja A., Zhao H., Yip K.P., Kwan S.Y., Onstad M., Sheng J., Zhu Y., Baluya D.L. (2020). ITLN1 modulates invasive potential and metabolic reprogramming of ovarian cancer cells in omental microenvironment. Nat. Commun..

[27] Dezso Z., Oestreicher J., Weaver A., Santiago S., Agoulnik S., Chow J., Oda Y., Funahashi Y. (2014). Gene expression profiling reveals epithelial mesenchymal transition (EMT) genes can selectively differentiate eribulin sensitive breast cancer cells. PLoS ONE.

[28] Marchion D.C., Cottrill H.M., Xiong Y., Chen N., Bicaku E., Fulp W.J., Bansal N., Chon H.S., Stickles X.B., Kamath S.G. (2011). BAD phosphorylation determines ovarian cancer chemosensitivity and patient survival. Clin. Cancer Res..

[29] Tai H., Wu Z., Sun S., Zhang Z., Xu C. (2018). FGFRL1 Promotes Ovarian Cancer Progression by Crosstalk with Hedgehog Signaling. J. Immunol. Res..

[30] Srinivasan S., Su M., Ravishankar S., Moore J., Head P., Dixon J.B., Vannberg F. (2017). TLR-exosomes exhibit distinct kinetics and effector function. Sci. Rep..

[31] Ingemarsdotter C.K., Tookman L.A., Browne A., Pirlo K., Cutts R., Chelela C., Khurrum K.F., Leung E.Y., Dowson S., Webber L. (2015). Paclitaxel resistance increases oncolytic adenovirus efficacy via upregulated CAR expression and dysfunctional cell cycle control. Mol. Oncol..

[32] Leung C.S., Yeung T.L., Yip K.P., Pradeep S., Balasubramanian L., Liu J., Wong K.K., Mangala L.S., Armaiz-Pena G.N., Lopez-Berestein G. (2014). Calcium-dependent FAK/CREB/TNNC1 signalling mediates the effect of stromal MFAP5 on ovarian cancer metastatic potential. Nat. Commun..

[33] Yu X., Vazquez A., Levine A.J., Carpizo D.R. (2012). Allele-specific p53 mutant reactivation. Cancer Cell.

[34] Zeller C., Dai W., Steele N.L., Siddiq A., Walley A.J., Wilhelm-Benartzi C.S., Rizzo S., van der Zee A., Plumb J.A., Brown R. (2012). Candidate DNA methylation drivers of acquired cisplatin resistance in ovarian cancer identified by methylome and expression profiling. Oncogene.

[35] Song H., Kwan S.Y., Izaguirre D.I., Zu Z., Tsang Y.T., Tung C.S., King E.R., Mok S.C., Gershenson D.M., Wong K.K. (2013). PAX2 Expression in Ovarian Cancer. Int. J. Mol. Sci..

[36] Guo Y., Nemeth J., O’Brien C., Susa M., Liu X., Zhang Z., Choy E., Mankin H., Hornicek F., Duan Z. (2010). Effects of siltuximab on the IL-6-induced signaling pathway in ovarian cancer. Clin. Cancer Res..

[37] Mok S.C., Bonome T., Vathipadiekal V., Bell A., Johnson M.E., Wong K.K., Park D.C., Hao K., Yip D.K., Donninger H. (2009). A gene signature predictive for outcome in advanced ovarian cancer identifies a survival factor: Microfibril-associated glycoprotein 2. Cancer Cell.

[38] Li M., Balch C., Montgomery J.S., Jeong M., Chung J.H., Yan P., Huang T.H., Kim S., Nephew K.P. (2009). Integrated analysis of DNA methylation and gene expression reveals specific signaling pathways associated with platinum resistance in ovarian cancer. BMC Med. Genom..

[39] Konstantinopoulos P.A., Fountzilas E., Pillay K., Zerbini L.F., Libermann T.A., Cannistra S.A., Spentzos D. (2008). Carboplatin-induced gene expression changes in vitro are prognostic of survival in epithelial ovarian cancer. BMC Med. Genom..

[40] Kulbe H., Chakravarty P., Leinster D.A., Charles K.A., Kwong J., Thompson R.G., Coward J.I., Schioppa T., Robinson S.C., Gallagher W.M. (2012). A dynamic inflammatory cytokine network in the human ovarian cancer microenvironment. Cancer Res..

[41] Cheadle C., Nesterova M., Watkins T., Barnes K.C., Hall J.C., Rosen A., Becker K.G., Cho-Chung Y.S. (2008). Regulatory subunits of PKA define an axis of cellular proliferation/differentiation in ovarian cancer cells. BMC Med. Genom..

[42] Buys T.P., Chari R., Lee E.H., Zhang M., MacAulay C., Lam S., Lam W.L., Ling V. (2007). Genetic changes in the evolution of multidrug resistance for cultured human ovarian cancer cells. Genes. Chromosomes Cancer.

[43] Bosotti R., Locatelli G., Healy S., Scacheri E., Sartori L., Mercurio C., Calogero R., Isacchi A. (2007). Cross platform microarray analysis for robust identification of differentially expressed genes. BMC Bioinform..

[44] Spillman M.A., Manning N.G., Dye W.W., Sartorius C.A., Post M.D., Harrell J.C., Jacobsen B.M., Horwitz K.B. (2010). Tissue-specific pathways for estrogen regulation of ovarian cancer growth and metastasis. Cancer Res..

[45] McCabe A., Zaheed O., McDade S.S., Dean K. (2023). Investigating the suitability of in vitro cell lines as models for the major subtypes of epithelial ovarian cancer. Front. Cell Dev. Biol..

[46] Ben-David U., Siranosian B., Ha G., Tang H., Oren Y., Hinohara K., Strathdee C.A., Dempster J., Lyons N.J., Burns R. (2018). Genetic and transcriptional evolution alters cancer cell line drug response. Nature.

[47] Li Q., Li Z., Luo T., Shi H. (2022). Targeting the PI3K/AKT/mTOR and RAF/MEK/ERK pathways for cancer therapy. Mol. Biomed..

[48] Cobb L., Gershenson D. (2023). Novel therapeutics in low-grade serous ovarian cancer. Int. J. Gynecol. Cancer.

[49] Cheaib B., Auguste A., Leary A. (2015). The PI3K/Akt/mTOR pathway in ovarian cancer: Therapeutic opportunities and challenges. Chin. J. Cancer.

[50] Lahtinen A., Lavikka K., Virtanen A., Li Y., Jamalzadeh S., Skorda A., Lauridsen A.R., Zhang K., Marchi G., Isoviita V.M. (2023). Evolutionary states and trajectories characterized by distinct pathways stratify patients with ovarian high grade serous carcinoma. Cancer Cell.

