# Supplementary material for: Patient-Representative Cell Line Models in a Heterogeneous Disease: Comparison of Signaling Transduction Pathway Activity Between Ovarian Cancer Cell Lines and Ovarian Cancer
Source: Cancers (Basel). 2024 Dec 2;16(23):4041. doi: 10.3390/cancers16234041 (PMC11640608; doi:10.3390/cancers16234041)

S5. Signal transduction pathway scores of cell lines grouped by cluster. AR= Androgen Receptor, ER= Estrogen Receptor, HH= Hedgehog, NF-κB= Nuclear factor kappa-light-chain-enhancer of activated B cells, TGF-β= Transforming Growth Factor- Bèta.

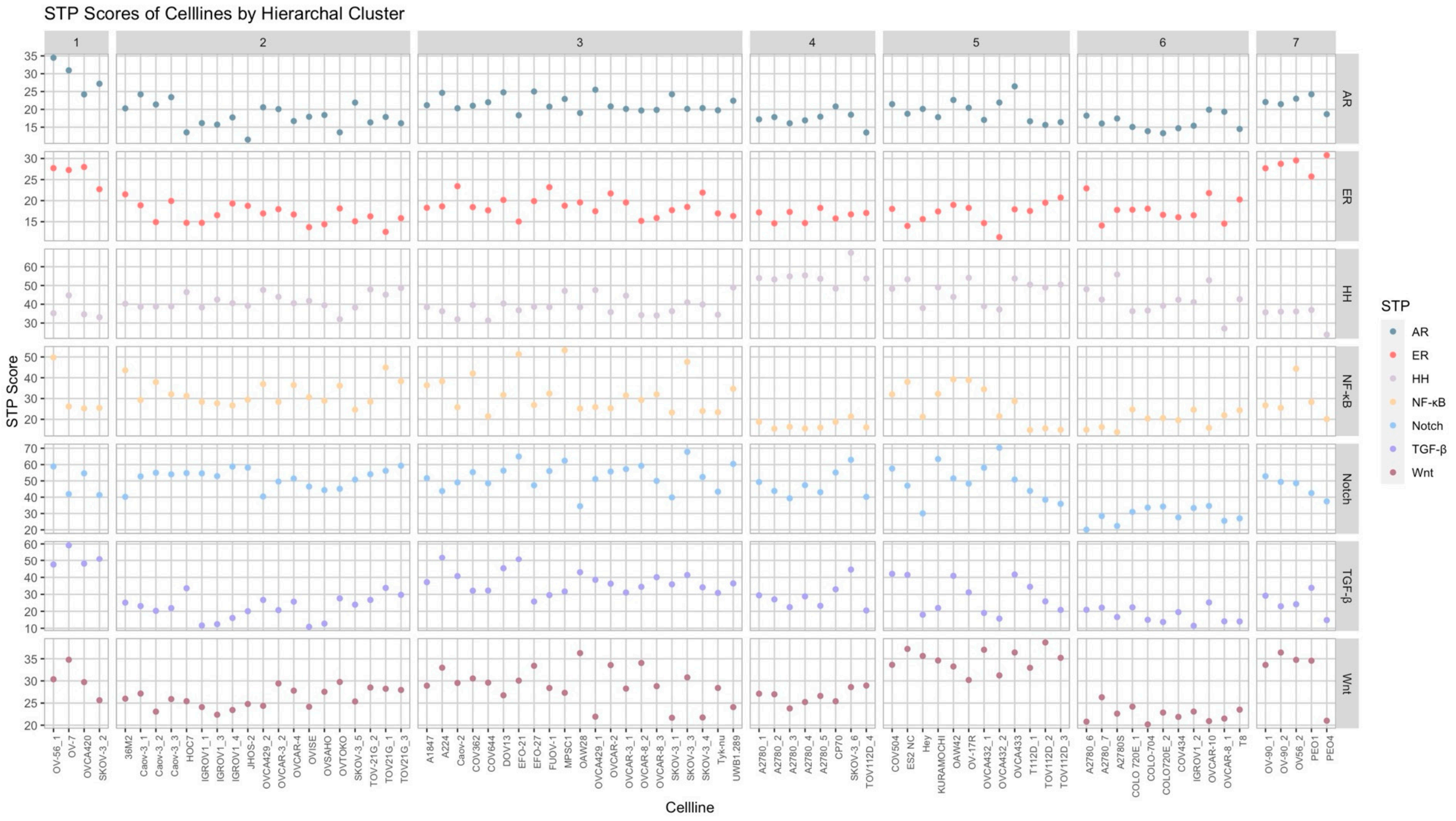

Supplement: Supplementary file 1 [file cancers-16-04041-s001.zip › Figure S5.pdf]
