# Supplementary material for: Patient-Representative Cell Line Models in a Heterogeneous Disease: Comparison of Signaling Transduction Pathway Activity Between Ovarian Cancer Cell Lines and Ovarian Cancer
Source: Cancers (Basel). 2024 Dec 2;16(23):4041. doi: 10.3390/cancers16234041 (PMC11640608; doi:10.3390/cancers16234041)
Supplement: Supplementary file 1 [file cancers-16-04041-s001.zip › Supplementary File S1.pdf]

**Supplementary File S1—Analysis by clusters of Tothill et al.**

## 1.Introduction

Based on the gene expression profile, serous and endometrioid ovarian carcinomas can be classified into six molecular subtypes as described by Tothill et al. [1]. Clinical outcome was shown to be related to the molecular subtype. In a similar manner tumor profiling by functional signal transduction pathway (STP) activity, taking not only the known genotype but also the phenotype into account, might have additional biological and clinical value.

## 2. Methods

### *2.1 K-means clustering*

We conducted k-means clustering analysis based on STP activity scores of the eligible cell lines. Since this did not result in stable clusters, we used the least squares method to classify each cell line in a specific predefined cluster from the publication of Tothill et al. [1]. The six molecular clinical subtypes produced by Tothill et al. were based on gene expression profiling results of OC samples through K-means clustering and class prediction. Using the GSE9891 dataset containing the Affymetrix microarray data from the clinical Tothill study we previously analyzed the STP activity of the patient-derived samples in each cluster [2]. Using the mean STP activity from each respective pathway in the Tothill dataset, we clustered the cell lines in the predefined Tothill clusters based on the activity score of the Androgen Receptor (AR), Estrogen Receptor (ER), Hedgehog (HH), Nuclear Factor kappa-light-chain-enhancer of activated B-cells (NF-κB), Notch, Transforming Growth Factor Beta (TGF-β) and Wnt STPs.

## 3. Results

### *3.1 STP activity*

Based on the mean STP activity, for each analyzed STP, of each of the six Tothill clusters, cell line samples were divided using the least squares method. Cell line samples were included in cluster 1, 3,4 and 5. There were no cell line samples included in cluster 2 and 6. The STP activity scores of individual cell line samples in their respective clusters are shown in Figure S1. Four cell lines were included in cluster 1. In this cluster, all cell line samples expressed a significantly higher NF-κB and Notch activity compared to the other clusters ($p<0.001$). The TGF-β pathway was also significantly higher compared to cluster 4 and 5 ($p<0.05$). In cluster 3, a total of thirteen cell line samples were included. In this cluster there was a significantly higher AR, ER TGF- β and Wnt STP activity compared to cluster 4 and 5 ($p<0.001$). In cluster 4, thirteen cell line samples were included. In this cluster the lowest TGF-β pathway was observed compared to the other clusters ($p<0.05$). Compared to cluster 3, the ER, AR and Wnt pathway activity was significantly lower in cluster 4 ($p<0.01$). In cluster 5, a total of 52 cell line samples were included. This cluster contains the cell line samples that had the lowest NF-κB STP activity compared to all other clusters ($p<0.001$). The HH pathway was significantly higher in

this cluster compared to cluster 3 and 4. Data is visualized in box plots in Figure S2. Table S1 compares the STP activity for each Tothill cluster as described by van Lieshout et al. compared to the STP characteristics for each cluster of the clustered cell lines in the Tothill clusters [2].

**Figure S1.** Individual signal transduction pathway scores of cell lines grouped by Tothill cluster. AR= Androgen Receptor, ER= Estrogen Receptor, HH= Hedgehog, NFKb= Nuclear factor kappa-light-chain-enhancer of activated B cells., TGF-β= Transforming Growth Factor-Bèta.

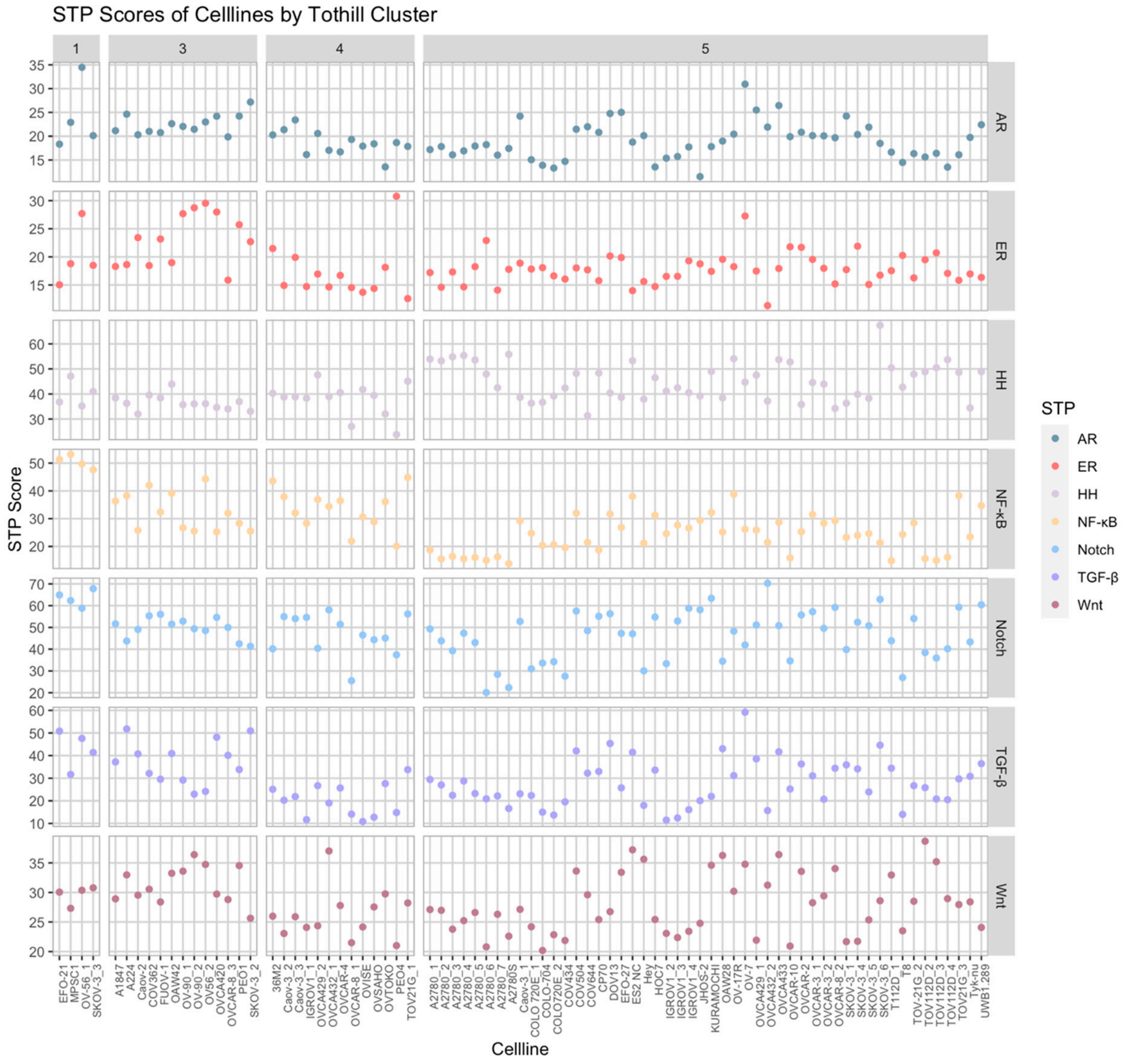

**Figure S2.** Comparison of signal transduction pathway activity by cluster defned by Tothill et al. AR= Androgen Receptor, ER= Estrogen Receptor, HH= Hedgehog, NFKb= Nuclear factor kappa-light-chain-enhancer of activated B cells., TGF-β=Transforming Growth Factor-Bèta.

Tothill et al. STP activity characteristics of cluster 1, 3, 4, and 5 as described by van Lieshout et al. (2020)

Cluster 1: high AR and TGF-β

Cluster 3: high ER and Wnt

Cluster 4: low TGF-β

Cluster 5: low AR and ER. High HH pathway.

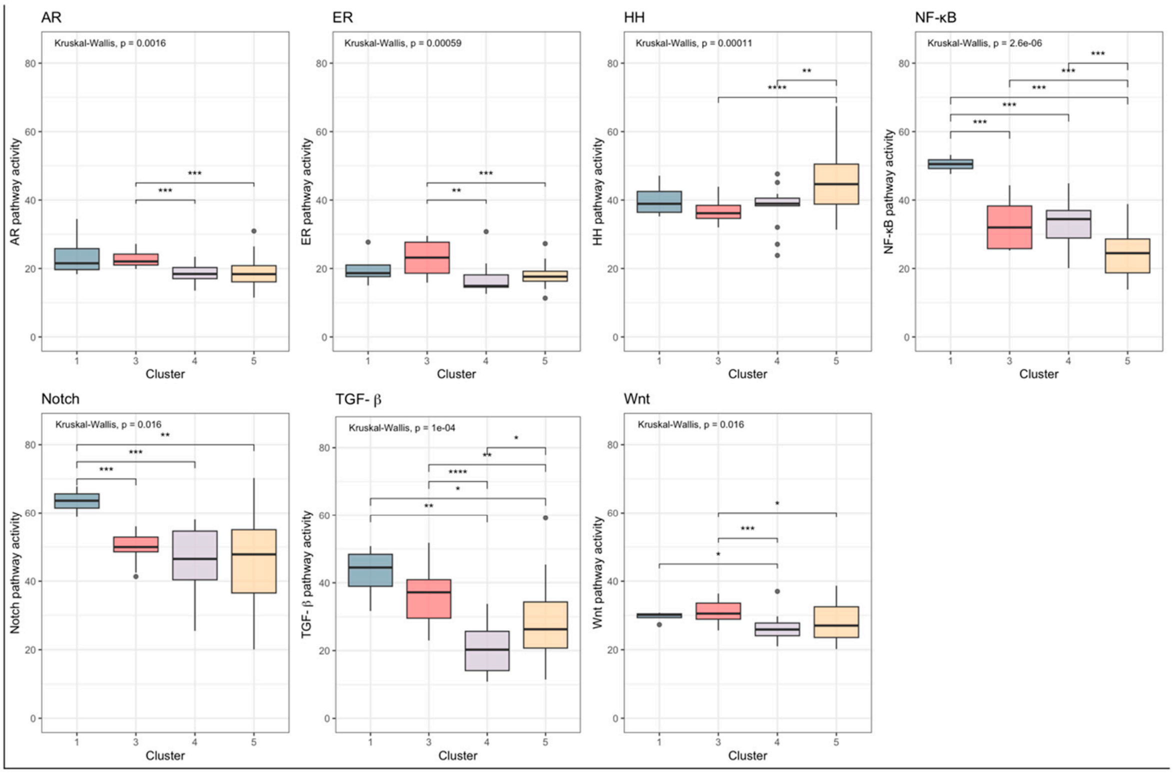

**Table S1.** Comparison of the signal transduction pathway (STP) profiles of the tumor tissue samples included in the Tothill clusters from the GSE9891 dataset and STP profiles of the cell lines included in these respective clusters.

| | **Cluster 1** | | **Cluster 3** | | **Cluster 4** | | **Cluster 5** | |
|---|---|---|---|---|---|---|---|---|
| | Tothill et al. | Cell line | Tothill et al. | Cell line | Tothill et al. | Cell line | Tothill et al. | Cell line |
| **AR** | High | - | - | Relatively high | - | - | Low | - |
| **ER** | - | - | High | Relatively high | - | - | Low | - |
| **HH** | - | - | - | - | - | - | High | Relatively high |
| **NF-κB** | - | High | - | - | - | - | - | Low |
| **Notch** | - | High | - | - | - | - | - | - |
| **TGF-β** | High | Relatively high | - | Relatively high | Low | Low | - | - |
| **Wnt** | - | | High | Relatively high | - | - | - | - |

*3.2 Tissue histology included in original Tothill cluster vs. cell line histology of origin*

The Tothill clusters were produced from tumor tissues of serous ovarian borderline tumors (SBOT), and serous (LGSOC or HGSOC) and endometrioid ovarian carcinomas (EOC). Cluster 1 in the Tothill paper was dominated by HGSC samples (99%). The cell lines included in this cluster were classified as ovarian serous adenocarcinoma (75%) and LGSOC. Cluster 3 contained ovarian tumors of low malignant potential, including SBOT and low-grade serous ovarian carcinomas (LGSOC), but also 21% of HGSC. This differed from the cell line histology of origin, which included HGSOC (23%), not further subtyped ovarian carcinoma (OC, 15.4%), and OCS. Both Cluster 4 and Cluster 5 from Tothill contained mainly HGSC, as well as endometrioid ovarian carcinoma (EOC) in Cluster 4. The cell lines in Cluster 4 were of EOC, HGSOC, OC, OCCC, and OCS origin. Cluster 5 was a mix of different (high grade) histological subtypes, except for ovarian tumors of low malignant potential (SBOT and LGSOC).

Table S2 compares the histology of the tumor tissue included in the Tothill clusters from the GSE09891 dataset and the histology of origin of the cell lines included in the respective clusters.

**Table S2.** Comparison of the proportions of histology of the tumor tissue included in the Tothill clusters from the GSE9891 dataset and the histology of origin of the cell lines included in the respective clusters.

| | **Cluster 1** | | **Cluster 3** | | **Cluster 4** | | **Cluster 5** | |
|---|---|---|---|---|---|---|---|---|
| | Tothill et al. | Cell line | Tothill et al. | Cell line | Tothill et al. | Cell line | Tothill et al. | Cell line |
| SBOT | - | - | 64% | - | - | - | - | - |
| LGSOC | - | 25% | 10.7% | - | - | - | - | - |
| EOC | 1% | - | - | - | 10.9% | 7.7% | - | 34.0% |
| HGSOC | 99% | - | 21.4% | 23% | 89.1% | 38.5% | 100% | 24.0% |
| MOC | - | - | - | - | - | - | - | 4.0% |
| OC | - | - | - | 15.4% | - | 7.7% | - | 5.0% |
| OCCC | - | - | - | - | - | 23.1% | - | 6.0% |
| OCS | - | 75% | - | 61.5% | - | 23.1% | - | 2.0% |
| OSCC | - | - | - | - | - | - | - | 2.0% |

SBOT= Serous Borderline Ovarian Tumor
EOC= Endometroid ovarian carcinoma
HGSOC= High Grade Serous ovarian carcinoma
LGSOC= Low-grade serous ovarian carcinoma
MOC= Mucinous ovarian carcinoma
OC= Ovarian Carcinoma
OCCC= Ovarian Clear Cell Carcinoma
OCS= Ovarian serous adenocarcinoma
OSCC= Ovarian small cell carcinoma, hypercalcemic type

## 4. Discussion

In this additional analysis we compared the STP activity of ovarian carcinoma cell line models to the six molecular subtypes produced by Tothill et al. The STP activity profiles of clinical (Tothill) and cell line samples in the same Tothill clusters showed some similarities but were not identical.

The histology of the original tumor of which the cell line was derived from, did not match the histology of the clinical samples included in the Tothill clusters (Table S1). This could partially be explained by missing information on histological subtype from the cell line of origin. Additionally, de novo mutations appear in cell line cultures, with possible effect of changes in the STP profile and therefore in the phenotype of the cell [3]. For example, a cell line originally derived from LGSOC, possibly would gain a phenotype of HGSOC due to changes in genotype and tumor microenvironment (cell culture).

## 5. Conclusions

In conclusion, cell lines divided over the clusters of Tothill et al. based on STP activity, do not seem like an accurate representation of the molecular subtypes described by Tothill et al. due to suboptimal matching of histological subtypes and differences in STP profiles.

## References

1. Tothill RW, Tinker AV, George J, et al. Novel molecular subtypes of serous and endometrioid ovarian cancer linked to clinical outcome. Clin Cancer Res. Aug 15 2008;14(16):5198-208. doi:10.1158/1078-0432.CCR-08-0196

2. van Lieshout L, van de Stolpe A, van der Ploeg P, Bowtell D, de Hullu J, Piek J. Signal Transduction Pathway Activity in High-Grade, Serous Ovarian Carcinoma Reveals a More Favorable Prognosis in Tumors with Low PI3K and High NF-kappaB Pathway Activity: A Novel Approach to a Long-Standing Enigma. Cancers (Basel). Sep 18 2020;12(9)doi:10.3390/cancers12092660
3. Ben-David U, Siranosian B, Ha G, et al. Genetic and transcriptional evolution alters cancer cell line drug response. Nature. Aug 2018;560(7718):325-330. doi:10.1038/s41586-018-0409-3
