# Supplementary figures and images for: Patient-Representative Cell Line Models in a Heterogeneous Disease: Comparison of Signaling Transduction Pathway Activity Between Ovarian Cancer Cell Lines and Ovarian Cancer
Source: Cancers (Basel). 2024 Dec 2;16(23):4041. doi: 10.3390/cancers16234041 (PMC11640608; doi:10.3390/cancers16234041)

**Figure S3.** Flowchart of GEO search and included cell lines in analysis.

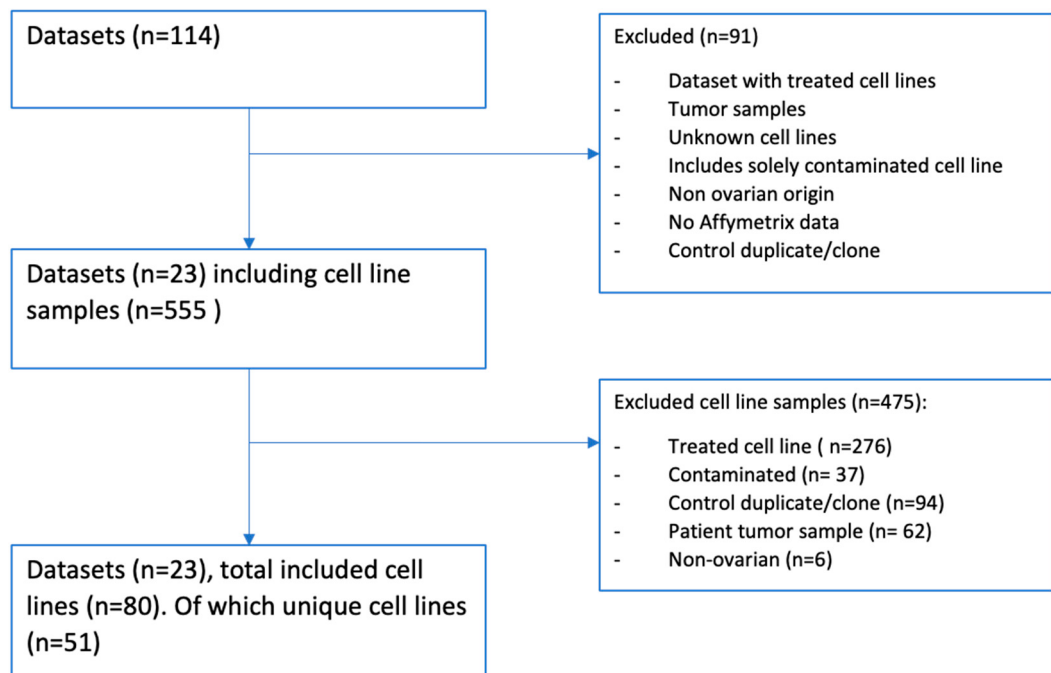

Supplement: Supplementary file 1 [file cancers-16-04041-s001.zip › Figure S3.pdf]
